# A Variable-Order Fractional Derivative Approach-Defined Zener Model and Its Application in Predicting the Compressive Creep Strain of Bamboo Scrimber

**DOI:** 10.3390/ma19071380

**Published:** 2026-03-31

**Authors:** Wenzijun Xiao, Songsong Sun, Xiaolin Gong, Jiahong Fu

**Affiliations:** 1 College of Automobile and Traffic Engineering, Nanjing Forestry University, Nanjing 210037, China; x1ao5985@163.com (W.X.);; 2 College of Engineering, Hangzhou City University, Hangzhou 310000, China

**Keywords:** creep, composite material, bamboo material, Caputo fractional derivative

## Abstract

Bamboo materials usually exhibit creep behavior under external loading conditions. This study conducted the compressive creep property research on bamboo scrimber, a commonly seen natural fiber-reinforced composite material. Firstly, the compressive creep strain data under various stress amplitudes were recorded on the basis of a four-step compressive load. Secondly, different Zener models were adopted in analyzing the recorded compressive creep process. Finally, a creep strain prediction method was proposed with the help of the verified model and the stress level-related empirical equations. The following main conclusions were drawn: for bamboo scrimber, the modified Zener model based on various-order Caputo fractional derivatives can provide precise expression in analyzing the creep performance of the material under compressive load than the traditional Zener model, as well as predict the creep strain increase property under other stress levels with a relatively shorter experimental period. Therefore, this method is valuable for promotion in modern industry.

## 1. Introduction

At present, various bamboo materials, such as bamboo scrimber, have been widely used in engineering applications [1]. Compared with raw bamboo, bamboo scrimber has much better mechanical properties and stability during the application process, which benefits its widespread use. As a result, its mechanical properties must be evaluated to guide its application in modern industry [2,3].

With this purpose, in recent years, numerous studies have been conducted by specialists in recent years. Huang applied various beam models to examine the strength properties of bamboo scrimber, establishing basic mechanical parameters [4,5]. Li conducted a tensile compression experiment with bamboo scrimber under different conditions to examine the mechanical constitutive relationship of bamboo scrimber and developed four models, which accurately characterize the stress–strain relationship at specified loading conditions [6]. Ma undertook research on the HCF of bamboo scrimber and established the value threshold of the high-cycle fatigue life. Further, residual stiffness of the specimen depends hugely on the stress level [7]. Shangguan examined the influence of heat treatment on the strength of the material and found that it can cause the induration of phenolic resin and even alter the crystallinity [8]. Yu studied the effect of the strength of bamboo scrimber because of the microstructural aspects, fiber–matrix interface properties, and the production process and realized that microstructural aspects can be maximized to realize a stronger material and that the interface property is the optimal mechanism to enhance the strengths of the material [9]. By considering the contribution of both the fiber and the matrix, Song analyzed the life of bamboo scrimber in terms of fatigue, which then encouraged the use of a two-parameter Weibull distribution function to model the stress–life correlation [10]. Yuan used hot oil that adsorbed on the fiber interface and was the heat treatment medium to investigate the impact of hot oil on the strength property of bamboo scrimber. In addition, the heat treatment bath time has to be sufficient [11].

The combination of cementing and pressing (cold and hot pressing) is currently the most widely used method of manufacturing bamboo scrimber. Viscoelastic mechanics theory leads to the belief that the composite materials formed as a result of this process tend to have obvious creep behavior in service. Thus, creep properties should be evaluated before application. In this field, several experts studied the creep performance of bamboo materials based on the selected viscoelastic models [12,13,14,15,16]. The results showed that the stress level effect on the creep behavior is obvious, as well as the temperature influence. In addition, the accelerated creep experiment is also an effective choice in researching this topic [17].

The abovementioned research clearly demonstrates that a corresponding creep experiment is an effective tool in the creep research of these bamboo materials [18,19]. This type of test can accurately evaluate the creep property of the selected materials according to the given standard but is associated with relatively high costs and long test periods, especially for long-term cases. However, during the experiment, the load amplitude should be controlled according to the given stress level. Therefore, any interruption will result in the failure of the experiment, which introduces additional risk to the experiment. Thus, quickly determining the creep of bamboo materials is important for their application.

In this paper, an accelerated creep test method is proposed for bamboo scrimber. A new kind of Zener model was built based on the variable-order fractional derivative approach. The creep test process was shortened by predicting the trend of the creep strain growth. In this way the risk and cost of the creep test can be reduced obviously due to the shorter period. The results showed that the proposed method can provide similar creep strain data with only half the experiment time; this makes it valuable for providing some theoretical guidance to design the safe and reliable application of bamboo scrimber.

## 2. Materials and Methods

### 2.1. Material and Specimen

The present paper targets the study of bamboo scrimber. The manufacturing process that would otherwise be employed is the hot-pressing process in the case of this composite material. The preliminary stage of this method is extraction of natural bamboo fibers obtained from raw bamboo material through use of a chemical (carbonization) and rolling procedure. Then the fibers were redried after the first gum dipping process. Finally, the fibers were rolled up and stamped into steel boxes that were of predetermined sizes and loaded with phenolic resin. The last step was to put the combination at the required temperature (130 °C) under pressure (20 MPa) in such a way that the fibers were reglued. Moso bamboo (approximately 4 or 5 years old) was the vital ingredient of the sample in this work, and the sample was made by the Tao Huajiang Company, Yiyang, China. The main information of the material is shown in Table 1.

**Figure 1:**
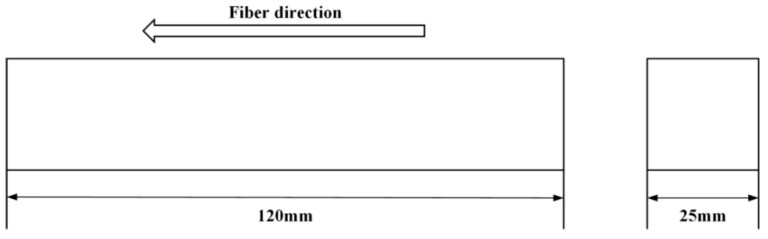
The main structural features of the test specimen in this paper.

At present, one of the most commonly seen application scenarios of bamboo scrimber is to build pillars in the construction and furniture industries. As a result of this, the load type is compressive. The specifications of the specimen are indicated in Figure 1. In order to be certain that the obtained result of the experiment could be considered reliable, ASTM standard 143-94 [20] was selected to make the corresponding cuboid sample.

### 2.2. The Test Method

**Figure 2:**
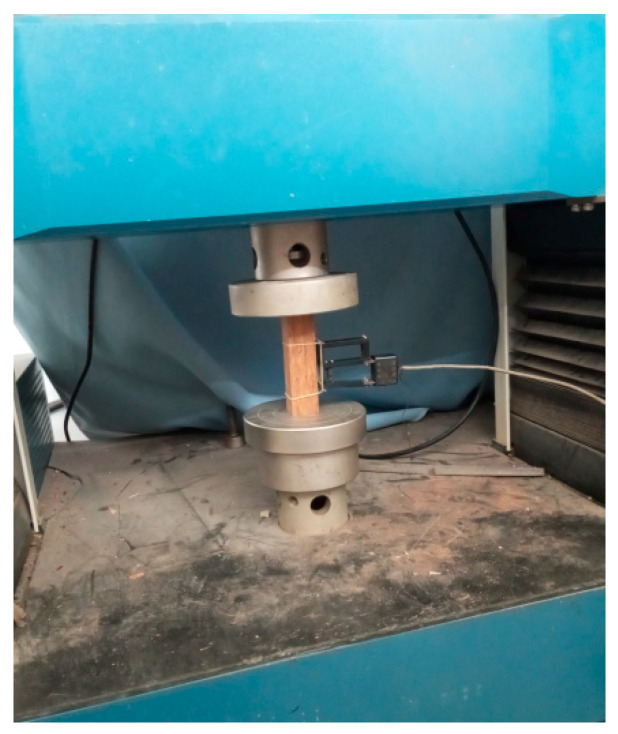
Compressive creep experimental equipment.

A widely applied method of research in the creep performance of the composite materials in modern studies is the standard test. Additionally, the creep strain or displacement developed during the creep process is the most common parameter used when measuring creep performance. The creep test corresponds to the test machine (Shenzhen Suns Technology Stock Co., Ltd., Shenzhen, China) illustrated in Figure 2, based on which it is possible to conclude that the strain is measured on the basis of the electronic extensometer (Reliant Technology LLC, Colorado Springs, CO, USA) attached to the sample. A study conducted earlier suggests that this strategy is appropriate compared to the strain gaps that should be used to determine compressive creep performance [12]. In this case, the number of the standard is ASTM D2990-17 [21,22].

**Figure 3:**
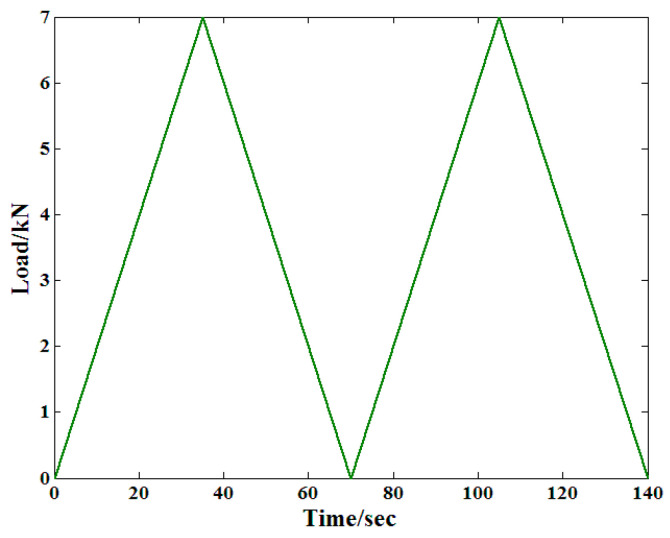
The pretreatment method for the specimen [23].

The test process in this study has a sampling frequency of 1 min. In the creep test process, a few things like minute spaces between the test equipment and the specimen can lead to a few measurement errors. Consequently, there must be preprocessing to ensure that the recorded strain is reliable. The main characteristics of the load are presented in Figure 3, because of which it may be concluded that the entire process was a set of two load cycles. Additionally, the rate of loading is usually constant throughout the entire process.

### 2.3. Compressive Creep Strain Prediction Method

In a previous related study, we analyzed the tensile creep strain of different samples under different stress levels [18]. The results revealed that the model parameters differed by sample and were difficult to analyze in detail, which can be attributed to the dispersiveness of the material itself. Therefore, studying the creep of a given sample based on the information obtained from other specimens is difficult, even if they were manufactured by the same technique.

**Figure 4:**
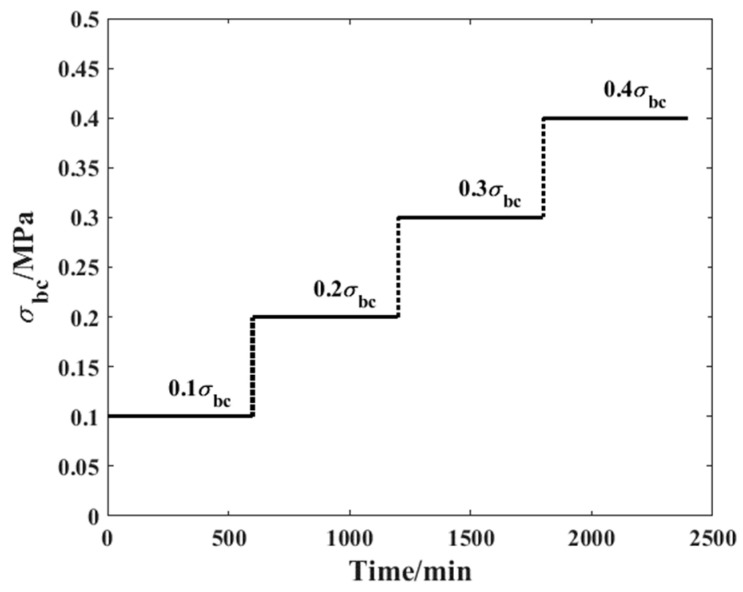
Load history of the compressive creep experiment.

On the other hand, according to previous study, when the commonly seen viscoelastic models (Burgers, Maxwell) were adopted in researching the creep performance of bamboo materials, the stress level effect on the main model parameters can accurately be expressed by the selected functions (linear, power or exponential types) [14,24]. Based on this fact, a prediction method was proposed as follows:

Step 1: A time-variant compressive load history was applied to a sample to study the related compressive creep process. Detailed information on the load history is shown in Figure 4.

Step 2: The creep behavior under different stress levels was analyzed based on a selected mechanical constitutive model. At the same time, the impact mechanism of the stress level effect on the model parameters was also analyzed if high enough accuracy had been provided in advance.

Step 3: Additional samples were selected for prediction. The creep strain under other lower stress levels and over longer durations was predicted with the help of values of the model parameters under higher stress conditions with shorter experimental times. In this way, the acceleration effect can be achieved based on the shorter experiment time with accurate prediction results.

### 2.4. Variable-Order Fractional Derivative Theory

**Figure 5:**
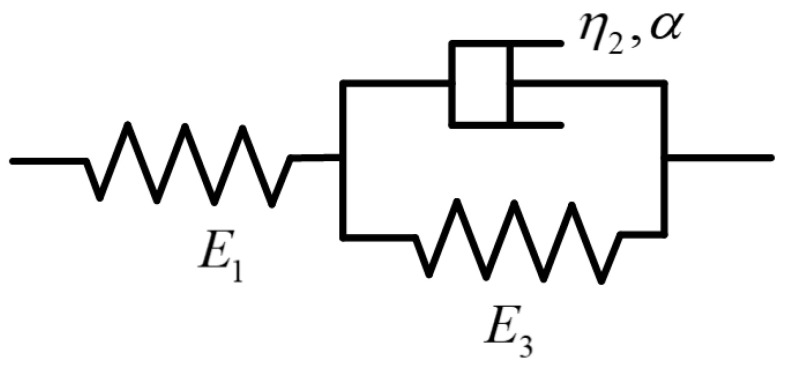
The Zener model and its main parts [26].

As mentioned in the preceding chapters, engineering bamboo materials usually exhibit pronounced creep behavior during the working process. Therefore, an appropriate viscoelastic mechanical constitutive model is necessary to analyze their stress–strain relationship. One of the most commonly used approaches is the Zener model. The primary aspect of the model composed of two spring bodies and one dashpot body is depicted in Figure 5. A spring is paralleled with the dashpot body, and this arrangement is partnered in series with the other spring [25].

In previous studies, the dashpot body has generally been considered the viscous part of the model in earlier studies. In case a load is exerted on the whole model, the stress–strain response can be expressed using:

(1) $$\sigma_{1}=\sigma_{2}+\sigma_{3}=\sigma_{0}$$

(2) $$\sigma_{1}\left(t\right)=E_{1}\varepsilon_{1}\left(t\right)$$

(3) $$\sigma_{3}\left(t\right)=E_{3}\varepsilon_{3}\left(t\right)$$

(4) $$\varepsilon_{3}\left(t\right)=\varepsilon_{2}\left(t\right)$$

where $\sigma_{0}$ denotes the total stress generated by the applied load; $\sigma_{1}$, $\sigma_{2}$, and $\sigma_{3}$ denote the stresses in the elastic spring body $E_{1}$, the Koeller dashpot body $\eta_{2}$, and the elastic spring body $E_{3}$, respectively; $\varepsilon_{1}$, $\varepsilon_{2}$, and $\varepsilon_{3}$ denote the corresponding strains in these components. The stress–strain relation of elastic spring bodies is established as shown in Equations (3) and (4). But with the dashpot, no universal definition exists that could be used to express the relationship. This relationship is traditionally defined as follows:

(5) $$\sigma_{2}\left(t\right)=\eta_{2}\frac{d\varepsilon_{2}\left(t\right)}{dt}$$

where $\;\eta_{2}$ is the viscosity coefficient of the model. Consequently, the response strain under a given stress $\sigma_{0}$ can be expressed as:

(6) $$\varepsilon_{1}\left(t\right)=\frac{\sigma_{0}}{E_{1}}$$

(7) $$\varepsilon_{2}\left(t\right)=\varepsilon_{3}\left(t\right)=\frac{\sigma_{0}}{E_{2}}(1-\exp(-\frac{E_{2}}{\eta_{2}}t))$$

(8) $$\varepsilon (t)=\varepsilon_{1}(t)+\varepsilon_{2}(t)=\varepsilon_{1}(t)+\varepsilon_{3}(t)=\frac{\sigma_{0}}{E_{1}}+\frac{\sigma_{0}}{E_{2}}(1-\exp(-\frac{E_{2}}{\eta_{2}}t))$$

Previously, the model parameters have usually been considered in past studies as the constants of the material without any dependence on the level of stress or any associated conditions. Among the studies conducted in recent years, however, some researchers have realized that the model is capable of having combined nonlinear and time-varying properties. At the same time, the fractional-order model is an effective one that has been taken into account in nonlinear and time-varying analysis. The element of the dashpot that is defined by this theory can be given as:

(9) $$\sigma_{2}=\eta_{2}\frac{d^{\alpha }\varepsilon_{2}\left(t\right)}{dt^{\alpha }}$$

where α is the order of the fractional derivative. In this paper, the Caputo model is chosen as the research tool. The definition of it is shown as [27]:

(10) $$D^{\alpha }f\left(t\right)=\frac{1}{\Gamma \left(1-\alpha \right)}\int_{0}^{t}\frac{f^{\prime }\left(\tau \right)}{{\left(t-\tau \right)}^{\alpha }}d\tau$$

where Γ is the gamma function. The corresponding output of the dashpot body can be expressed as:

(11) $$\begin{array}{cc} \sigma_{2} & =\eta \left(t\right)\frac{d^{\alpha }\varepsilon_{2}\left(t\right)}{dt^{\alpha }}\\ & =\eta \left(t\right)\int_{0}^{t}\frac{\varepsilon_{2}^{\prime }\left(\tau \right)}{\Gamma \left(1-\alpha \right){\left(t-\tau \right)}^{\alpha }}d\tau \end{array}$$

Figure 4 presents a load history, which demonstrates evident time-varying property. Based on our earlier study, the order of the fractional derivative would strongly depend on the level of stress applied when the level is incorporated to examine the creep characteristics of composite materials. Accordingly, when the Caputo model is applied in the study of the creep performance of bamboo scrimber, even the order of the fractional derivative will be dependent on the level of stress. The relationship is:

(12) $$\begin{array}{cc} \sigma_{2} & =\eta \left(t\right)\frac{d^{\alpha }\varepsilon_{2}\left(t\right)}{dt^{\alpha }}\\ & =\eta \left(t\right)\int_{0}^{t}\frac{\varepsilon_{2}^{\prime }\left(\tau \right)}{\Gamma \left(1-\alpha \right){\left(t-\tau \right)}^{\alpha }}d\tau \\ & \approx \sum_{i=0}^{k}\eta \left(t_{i+1}\right)\int_{i\mu }^{\left(i+1\right)\mu }\frac{\partial \varepsilon_{2}\left(\tau \right)}{\partial \tau }\frac{1}{\Gamma \left[1-\alpha \left(t_{i+1}\right)\right]}\frac{d\tau }{{\left(t_{k+1}-\tau \right)}^{\alpha \left(t_{i+1}\right)}} \end{array}$$

As shown in Equation (6), $\eta \left(t_{i+1}\right)$ and $\alpha \left(t_{i+1}\right)$ are the viscosity coefficient and the fractional derivative order (*i* is the number of the time step), which may be roughly estimated as material constants in this entry range assuming the level of stress applied does not vary. As Equation (13) indicates, two factors define the varying effect of the variable-order Caputo model with time: the present time and its time-dependent change. It leads to a stronger time-varying effect than traditional fractional derivative models, like the Riemann Liouville (R stands for Riemann, L for Liouville) model. Also, as specified in the load history, the time it takes to cycle between the levels of stress is the same (600 min). The viscoelastic property of the dashpot body corresponding to this theory can therefore be defined as:

(13) $$\begin{array}{cc} \sigma_{2} & \approx \sum_{i=0}^{k}\eta \left(t_{i+1}\right)\frac{\varepsilon_{2}\left(t_{i+1}\right)-\varepsilon_{2}\left(t_{i+1}\right)}{\mu }\int_{i\mu }^{\left(i+1\right)\mu }\frac{1}{\Gamma \left[1-\alpha \left(t_{i+1}\right)\right]}\frac{d\tau }{{\left(t_{k+1}-\tau \right)}^{\alpha \left(t_{i+1}\right)}}\\ & =\sum_{i=0}^{k}\eta \left(t_{i+1}\right)\frac{\varepsilon_{2}\left(t_{i+1}\right)-\varepsilon_{2}\left(t_{i+1}\right)}{\mu }\int_{\left(k-i\right)\mu }^{\left(k-i+1\right)\mu }\frac{1}{\Gamma \left[1-\alpha \left(t_{i+1}\right)\right]}\frac{d\varphi }{\varphi^{\alpha \left(t_{i+1}\right)}}\\ & =\sum_{i=0}^{k}\eta \left(t_{i+1}\right)\frac{\varepsilon_{2}\left(t_{i+1}\right)-\varepsilon_{2}\left(t_{i+1}\right)}{\mu }\int_{i\mu }^{\left(i+1\right)\mu }\frac{1}{\Gamma \left[1-\alpha \left(t_{i+1}\right)\right]}\frac{d\varphi }{\varphi^{\alpha \left(t_{i+1}\right)}}\\ & =\sum_{i=0}^{k}\eta \left(t_{i+1}\right)\frac{\varepsilon_{2}\left(t_{i+1}\right)-\varepsilon_{2}\left(t_{i+1}\right)}{\mu }\frac{1}{\Gamma \left[1-\alpha \left(t_{i+1}\right)\right]}\int_{i\mu }^{\left(i+1\right)\mu }\frac{d\varphi }{\varphi^{\alpha \left(t_{i+1}\right)}}\\ & =\sum_{i=0}^{k}\eta \left(t_{i+1}\right)\left[\varepsilon_{2}\left(t_{i+1}\right)-\varepsilon_{2}\left(t_{i+1}\right)\right]\frac{\mu^{-\alpha \left(t_{i+1}\right)}}{\Gamma \left[2-\alpha \left(t_{i+1}\right)\right]}\left[{\left(i+1\right)}^{1-\alpha \left(t_{i+1}\right)}-i^{1-\alpha \left(t_{i+1}\right)}\right] \end{array}$$

where *t* is the time quantum of the total loading spectrum and μ is the time quantum of an individual time step. They could be compared to one another as follows:

(14) t=(k+1)μ

The strain of the dashpot body and of the whole Kelvin–Voigt model can be analyzed according to this definition when the level of stress is known.

## 3. Results

### 3.1. Compressive Creep Results

**Figure 6:**
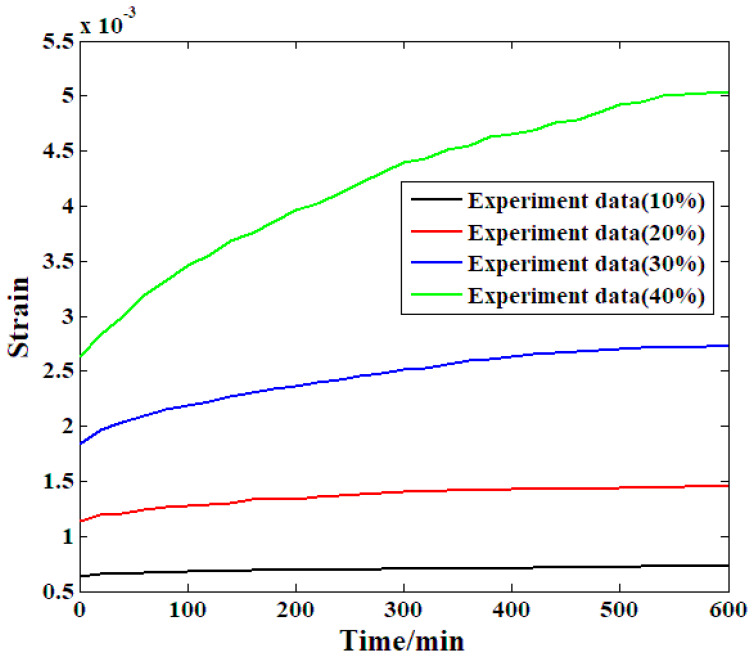
The recorded compressive creep strain of the primary samples.

In accordance with the research method mentioned above, the first step of the accelerated creep experiment is to analyze the compressive creep strain evolution under different stress levels. In this step, a sample was selected as the primary research object and subjected to proposed creep experiments according to the load history in Figure 4. This experiment provided the base data for further analysis. The detailed creep strain evolution process is shown in Figure 6. During the experiment process, no obvious crack or deformation has been found among the specimens.

The creep strain evolution recorded from the primary sample under all load stages is shown in Figure 6. The curve of the strain under the lowest stress level also has the lowest slope. The strain evolution at this stress level is generally steady. At relatively high stress conditions (the second stress level), the growth speed of the creep strain is steep at the beginning stage of the whole evolution process. In addition, the acceleration at this stress level is much greater than that at the previous stress level. After approximately 100 min, the increase in strain decelerates and the curve flattens. For the third experimental condition of this sample (30% of the compressive strength), the increase in strain markedly accelerates as the stress level increases. In addition, the increase in strain began to decelerate at approximately 460 min into the experiment. At the highest stress level, the increase in creep strain is also the greatest (the relative increase is nearly 90%). Moreover, the deceleration point of the strain growth rate appears nearly at the final stage of the whole process. Comparing the strain evolution recorded from this sample at all four stress levels yields two primary conclusions: The magnitude of the applied stress during the creep experiment directly correlates with the increase in creep strain and the duration of strain increase, i.e., deceleration begins at a later time. This can be explained by the microstructural and structural properties of the bamboo scrimber. Based on the production technique of the samples presented above, the bamboo scrimber is mainly made of bamboo fibers and phenolic resin. The creep of these two factors has a great influence on their nature. In general, a very large amount of variation would be possible between the rates of increase and decrease of the creep strain under the same level of stress and other parameters of mechanical properties, such that their level of Young’s modulus would not be the same, and this would make the interface of the two materials susceptible to shear stress where there is a dissimilarity in the materials. In the creep test at the first stage, the fiber and the matrix can resist creep deformation during the first level of deformation. In addition, their interstitial pores will not be destroyed. The strain accumulation relative to the higher displacement and the destruction of the pores is relative to the time of the experiment; the longer it is, the more can be destroyed as there is an inhomogeneity between the fiber and the matrix. This process in turn recombined the sample to form a new type; therefore, the growth characteristic of the strains was altered [28].

### 3.2. Model Analysis Results

**Figure 7:**
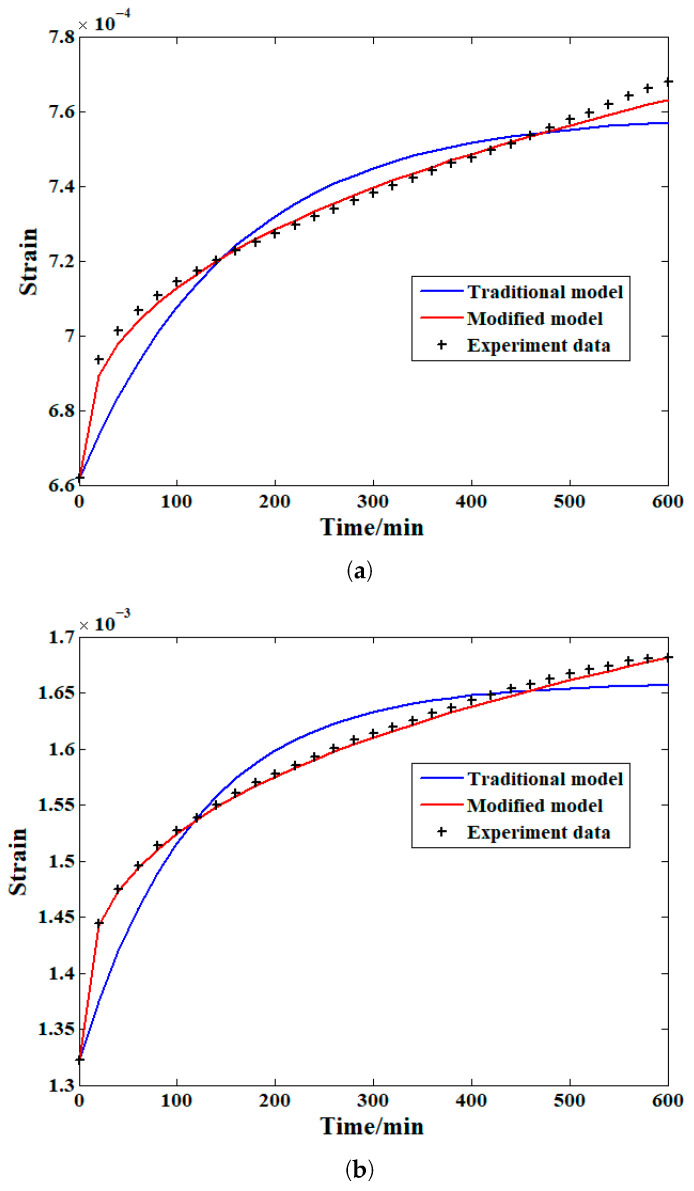

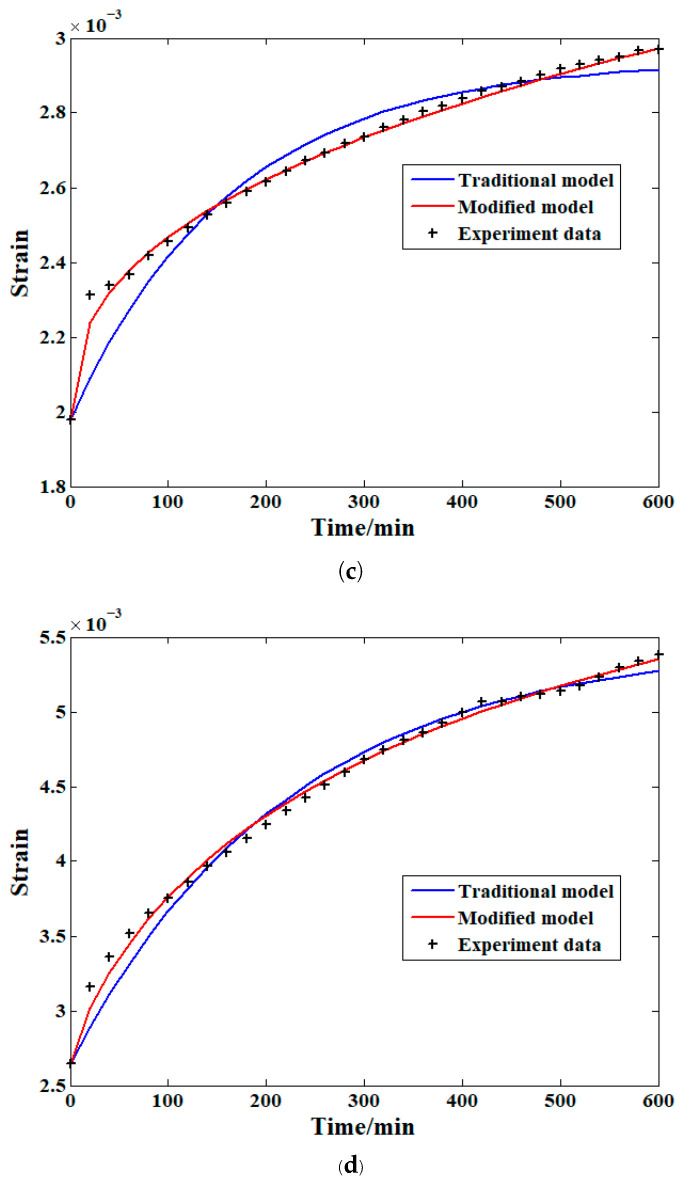
Fitting results based on both Zener models (**a**) at the first stress level; (**b**) at the second stress level; (**c**) at the third stress level; and (**d**) at the fourth stress level.

The practicability of the proposed model in the field of creep property research can be analyzed based on the strain evolution recorded in the previous section. To further strengthen the conclusions of this study, both the traditional and the newly proposed Zener models were selected to fit the time–strain history of the samples at all four stress levels. The Levenberg–Marquardt algorithm was selected in the fitting process. The analysis results of the experiment data according to this approach and different Zener models are shown in Figure 7.

As shown in Figure 7a, for the compressive creep test results under the first stress level condition, the fitting results and original experimental data markedly differed when the classical Zener model was chosen for researching the laws of growth. For the modified Zener model defined according to the proposed fractional derivative model, the fitting results are nearly the same as the experimental data. With respect to the analysis results on the basis of the second stress level shown in Figure 7b, a similar situation can be found, i.e., the modified Zener model also shows higher accuracy in analyzing the creep strain in this condition. For the fitting results obtained from the other two stress levels, although the accuracy of the fitting results provided by the traditional Zener model has been improved to a certain extent, the modified Zener model is also capable of providing satisfactory results.

Table 2 shows the correlation coefficient of the analysis results in all the cases based on both the traditional and variable-order Caputo fractional derivative-defined Zener models. Compared with the fitting results based on the traditional Zener model, the variable-order Caputo fractional derivative-defined Zener model can provide much higher accuracy for the fitting results in all cases (more than 99%), especially when the applied stress level is relatively low. Moreover, the accuracy provided by the modified model at other stress levels remains high. Generally, this model is more suitable for analyzing compressive creep in this field.

In order to carry out the deep analysis of the effect caused by the stress amplitude on the material creep behavior, the fitted results must be quantitatively analyzed. The influence of the stress level on the elastic properties of bamboo scrimber was studied in detail in a previous study [29]. Therefore, in this study, the influence of the stress level on the viscoelastic properties of the material was analyzed. In accordance with the principal members of the Zener model, the Kelvin body is also used to define the viscoelasticity of the model. In addition, the order of the fractional function also impacts the model’s viscoelastic property.

In this study, two commonly seen functions were chosen to analyze the impact generated by the stress level on the model parameters: a power function and an exponential function. The detailed information of these two models is shown in Table 3.

**Figure 8:**
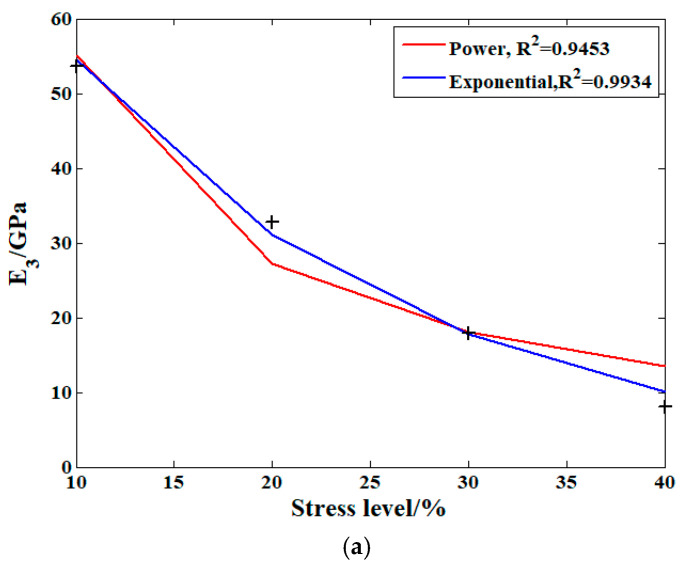

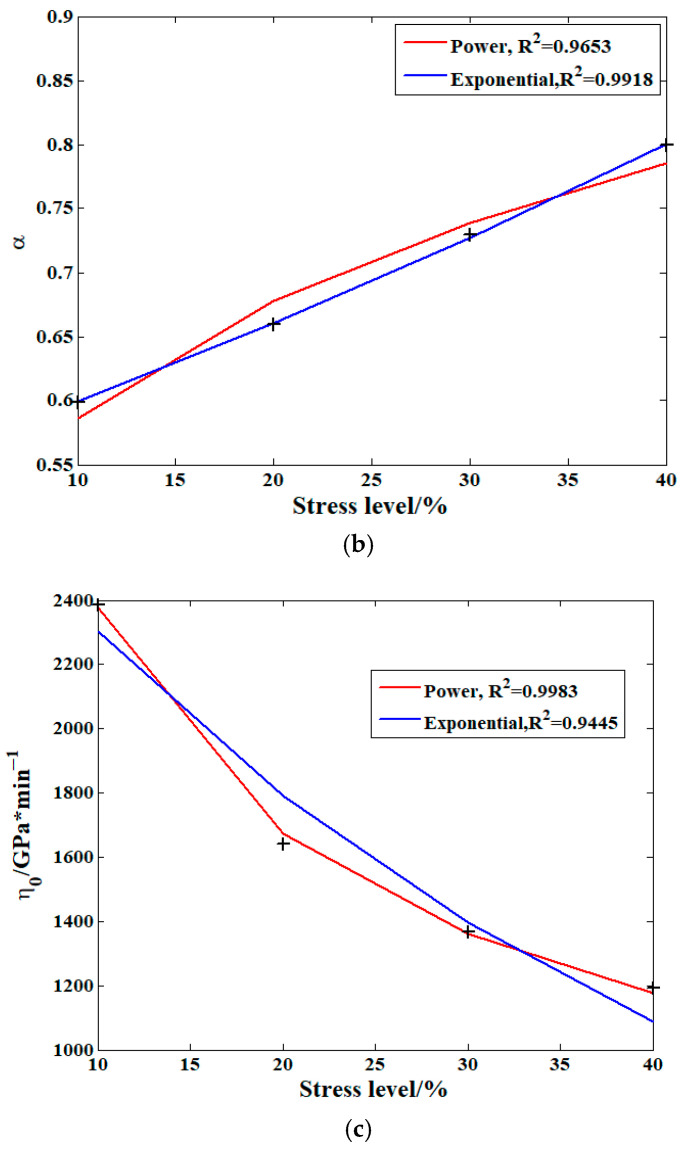
The effect of the stress level on the main parameters: (**a**) the elasticity modulus $E_{3}$; (**b**) the order; and (**c**) the viscosity coefficient $\eta_{2}$.

As shown in Table 3, where ***P*** is the value of the model parameter, ***A***, ***B***, ***C*** and ***D*** can be considered as constants once the specimen has not been changed, and σ is the stress amplitude provided by the creep test load condition. Based on these two functions, the stress level influence on the parameters of the proposed model can be analyzed. The analysis conclusions are shown in Figure 8.

As shown in Figure 8, the symbols + represent the original experiment data points. For the effect provided by the stress amplitude on the main model parameters, the exponential function can accurately reflect this influence on the order of the fractional derivative, as well as the elastic modulus of the Kelvin body. In addition, the power function is better for analyzing this effect on the viscosity coefficient.

### 3.3. Prediction Results

These relationships can be used to describe the creep strain evolution of a sample at other stress levels and aid the design of materials under variable stress conditions. To further analyze the creep strain growth property of bamboo scrimber at different stress levels, the relative increase in the creep strain was adopted for a more detailed evaluation. This parameter can be defined as follows:

(15) $$\xi =\left|\frac{\varepsilon (t)-\varepsilon_{0}}{\varepsilon_{0}}\right|\times 100\%$$

where ξ is the relative increase, ε(t) is the creep strain evolution, and $\varepsilon_{0}$ is the recorded starting value of the creep strain. Table 4 shows the relative increase values at different time nodes for all four stress levels, which show that the level of stress directly correlates with the rate of strain increase. For the lower stress levels (10% and 20% of the compressive strength), the final values of the relative increase in the creep strain are no more than 30%. With respect to the higher stress levels, this degree of relative increase accounts for only approximately one-quarter of the entire creep process.

**Figure 9:**
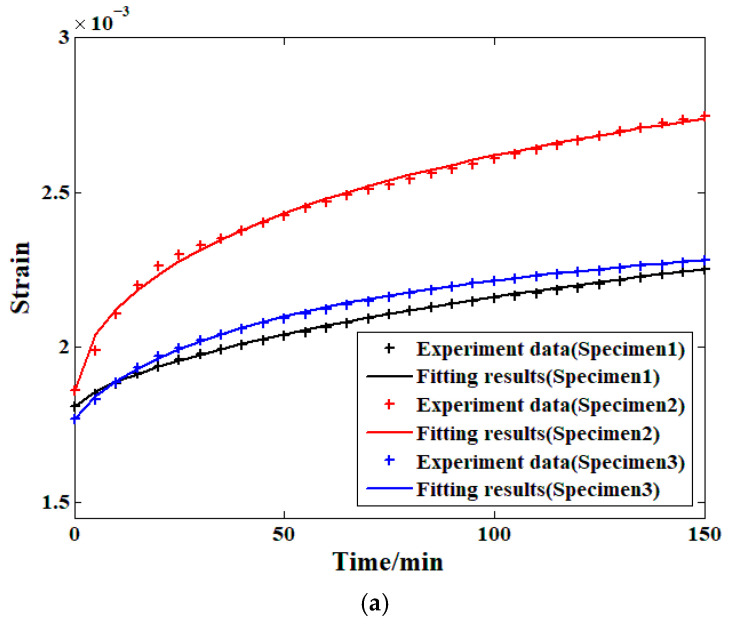

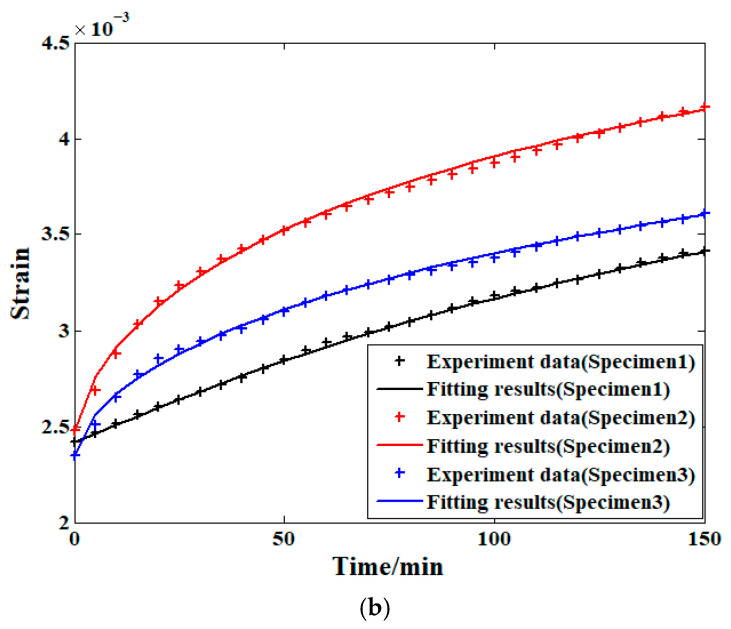
The analysis results of all three samples: (**a**) 30% stress level; (**b**) 40% stress level.

On this basis, the prediction approach in this paper is proposed in the following steps. Firstly, a two-step load spectrum created based on relatively higher stress levels (30% and 40% of the compressive strength) with a relatively shorter period was applied to the specimen to record the strain evolution process under these load conditions. Secondly, the model parameter values were determined by fitting the recorded data. Finally, the model parameter values under other lower stress levels (10% and 20% of the compressive strength) were determined based on the verified stress level functions in Figure 8. In addition, three groups of samples were selected as the prediction objects to ensure the universality of the proposed method. The research results of the first step are shown in Figure 9.

From the information provided by Figure 9, it is clear that when the modified Zener model was selected to analyze the creep strain of all three samples at these two stress levels, the accuracy was sufficiently high in all cases (the relative difference between the fitting results and the original experimental data at each time node is less than 1%). In addition, each load spectrum lasts for 150 min. Thus, the fitting results can be treated as the basis for further prediction research. The results are shown in Table 5.

Table 6 shows the model parameter values determined by the mentioned fitting approach above, as well as the functions based on the determined parameters; in this way, the model parameter estimation at any specified stress level can be determined.

**Figure 10:**
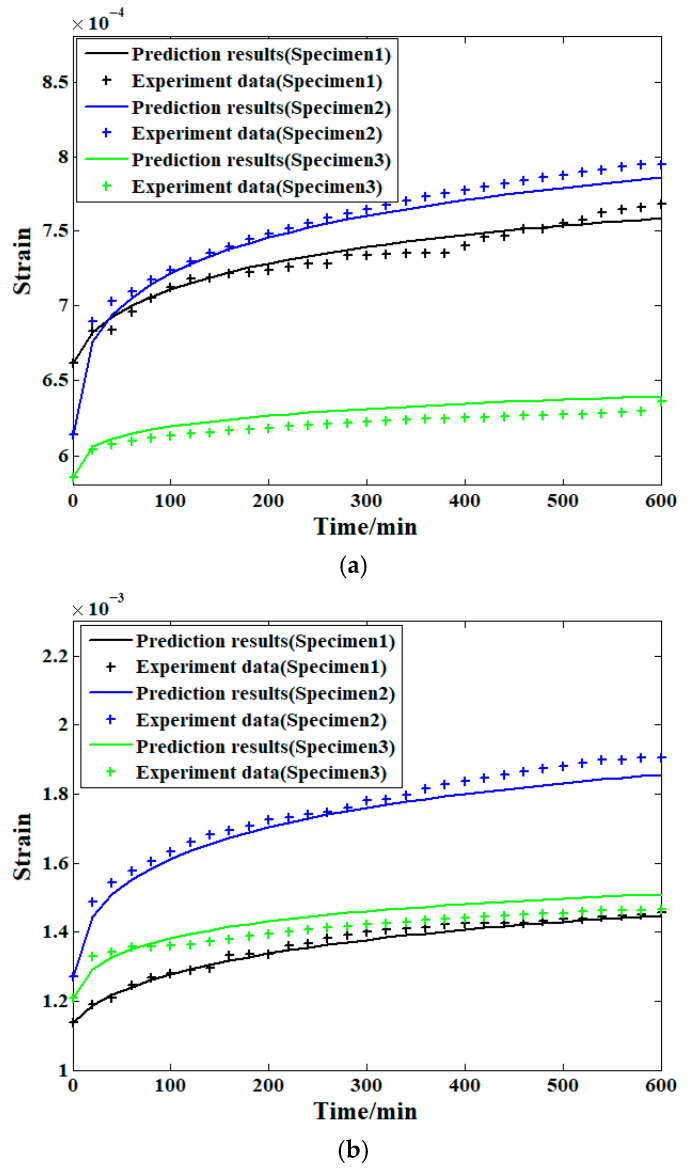

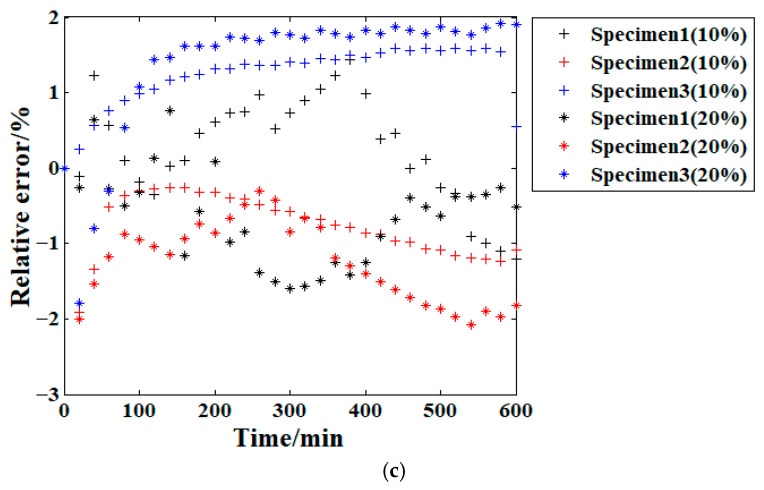
Forecasting results of the compressive creep strain: (**a**) 10% stress level; (**b**) 20% stress level; (**c**) residual curves of all the cases.

As introduced above, the compressive creep strain behavior of the samples at stress levels of 10% and 20% was selected as the prediction object. In addition, the verification work was conducted by the corresponding two-step load spectrum. The prediction results of all three samples are shown in Figure 10, which shows that the predicted strains of all these three samples at these two stress levels are similar to the actual result obtained through the experiment. In addition, the short-term compressive creep experiments at both stress levels lasted for 300 min in all (150 min for each stress level). For the prediction cases, the process lasted 600 min. In other words, the prediction approach can provide nearly the same experimental data within only half of the experimental time; this acceleration reduces the corresponding time and cost, as well as the risk of test failure. Meanwhile, although the model parameter estimations obtained from different samples are clearly random, the dispersion of the data does not affect the prediction accuracy. This can be explained by the fact that the same prediction object in each prediction case is just the supplier of the training data obtained through higher stress level experiments. Based on this, the elimination of the dispersion effects of the material itself can be achieved.

## 4. Conclusions

Creep is a critical factor in guiding the design of safe and reliable natural fiber-reinforced materials such as bamboo scrimber. In this study, a corresponding study was conducted on the compressive performance of the material. A time-varying compressive load defined according to different stress levels was applied to the specimen. Subsequently, different Zener models were chosen for comparative study on the topic. The results revealed that when the compressive load is acting on the bamboo scrimber, the rate of compressive creep strain increase is clearly influenced by the stress amplitude applied to it. The increase in creep under the four given stress levels gradually slowed after an initial rapid growth stage; the higher the applied stress level, the later this inflection occurred, which can be attributed to the microstructural features of the material.

Due to the effect of the fractional derivative approach, the newly developed Zener model can precisely express the creep behavior of bamboo scrimber. The proposed prediction method in this paper can accurately predict the creep strain growth trend within the following process. This makes it possible to take the actual experiment instead to reduce cost and risk, and it can thus be adopted in modern industry.

In this paper, the accelerated effect is achieved by predicting the creep strain growth property. The time-varying load may sometimes result in the data fluctuation of the strain test results. On the other hand, according to previous study, the temperature is an important factor in affecting the creep performance of bamboo materials. Thus, whether the accelerated creep test method proposed in this paper can also be useful under high temperature conditions is still unknown.

## Figures and Tables

**Table 1:** The basic information of the material.

Parameter	Value
Density	1.28 T/m^3^
Water content	Less than 6%
Fiber percentage	Over 70%
Formaldehyde emission rate	Less than 0.5 mg/L

**Table 2:** $R^{2}$ of the fitting results based on both models.

Stress Level	Traditional	Modified
10%	0.8536	0.9954
20%	0.9917	0.9958
30%	0.9922	0.9965
40%	0.9984	0.9949
Mean	0.9589	0.9957

**Table 3:** The definition of different relationship functions.

Model Type	Definition
Power	$P=A\sigma^{B}$
Exponential	P=Cexp(Dσ)

**Table 4:** Relative increase in the sample at different time nodes and stress levels.

Time	10%	20%	30%	40%
150 min	8.6	13.7	21.2	39.7
300 min	10.7	22.4	35.1	63.3
450 min	12.7	25.3	44.3	77.8
600 min	16	28.1	48.1	91.1

**Table 5:** The fitting results of the three samples under both stress levels.

Specimen Number	30%	40%
1	0.9918	0.9981
2	0.9957	0.9936
3	0.9984	0.9975
Mean	0.9953	0.9964

**Table 6:** Model parameter analysis results.

Number	Parameter	30%	40%	Function
1	Order	0.818	0.9	C=0.6142,D=0.01217
	Elastic modulus	13.8 GPa	7.06 GPa	C=104.1,D=−0.08568
	Viscosity coefficient	$2492\;\mathrm{GPa}\cdot \min^{-1}$	$2345\;\mathrm{GPa}\cdot \min^{-1}$	A=4841,B=−0.2103
2	Order	0.73	0.8	C=0.5547,D=0.01166
	Elastic modulus	17.9 GPa	8.07 GPa	C=195.2,D=−0.1015
	Viscosity coefficient	$1396\;\mathrm{GPa}\cdot \min^{-1}$	$1193\;\mathrm{GPa}\cdot \min^{-1}$	A=6203,B=−0.4087
3	Order	0.61	0.7	C=0.4037,D=0.01753
	Elastic modulus	13.1 GPa	8.34 GPa	C=49.4,D=−0.05663
	Viscosity coefficient	$569\;\mathrm{GPa}\cdot \min^{-1}$	$566\;\mathrm{GPa}\cdot \min^{-1}$	A=606,B=−0.0199

## Data Availability

The original contributions presented in this study are included in the article. Further inquiries can be directed to the corresponding author.
